# Associations between executive functions, coaches’ evaluation, and performance development in youth soccer

**DOI:** 10.1038/s41598-025-33159-4

**Published:** 2025-12-22

**Authors:** S. Knöbel, M. L. Reinhard, A. Borchert, N. Gatzmaga, L. Musculus, F. Lautenbach

**Affiliations:** 1 Institute of Sport Science, Sport Psychology, Humboldt-Universität zu Berlin, Philippstraße 13, 10115 Berlin, Germany; 2 Faculty of Sport Science, Chair of Sport Psychology, Universität Leipzig, Jahnallee 59, 04109 Leipzig, Germany; 3 Institut für Sportwissenschaft, Arbeitsbereich für Sportpsychologie und Methodenlehre, Universität Tübingen, Tübingen, Germany; 4 VfB Stuttgart 1893 AG, Mercedesstraße 109, 70372 Stuttgart, Germany; 5 RasenBallsport Leipzig GmbH, Cottaweg 3, 04177 Leipzig, Germany; 6 Deutscher Fußball-Bund e.V., Kennedyallee 274, 60528 Frankfurt/Main, Germany; 7 Department Performance Psychology, Institute of Psychology, German Sport University Cologne, Am Sportpark Müngersdorf 6, 50933 Cologne, Germany; 8 Institute of Sport and Sport Science, Sportpsychology, TU Dortmund, Otto-Hahn-Str. 3, 44227 Dortmund, Germany

**Keywords:** Cognition, Diagnostics, Elite soccer, Talent development, Talent prognosis, Human behaviour, Ageing

## Abstract

Understanding the role of executive functions (EFs) in sports performance is a central focus for practitioners and researchers in sports psychology, particularly in soccer. Prior studies suggest links between EFs and elite performance or game-specific skills, increasing their consideration in talent identification and development. Yet, the validity and predictive value of EF measures remain uncertain. This study examines the relationship between inhibition, cognitive flexibility and soccer performance development in a sample of youth elite players (*M*_age_ = 15.17, *SD*_age_ = 1.3). Specifically, we investigate (i) associations with coaches’ evaluations of future potential (*n* = 74), (ii) age-related development (*n* = 34), and (iii) the predictive value of EFs for senior performance levels (*n* = 69). Multinomial logistic regression analyses revealed no significant evidence that EFs explain elite soccer performance (for (i) all *p* ≥ .125; for (iii): all *p* ≥ .058). Multilevel analyses showed cognitive flexibility following age-related developmental trends similar to the general population (ii). These findings question whether a robust link between EFs and performance ratings exists. Given the small sample size, this study emphasizes the need for further, more nuanced research on the predictive value of EFs in the context of talent identification and development.

## Introduction

Researchers and practitioners emphasize the multifaceted and complex performance demands in soccer. This complexity has driven an increasing integration of scientific analysis into the applied work in recent years to identify and investigate predictors of elite performance with the overarching aim to optimize talent identification and development^1,2^. Psychological factors, particularly cognitive skills, have become a key focus^2–4^. Among others, the core executive functions (EF) inhibition, working memory and cognitive flexibility are considered as potential predictors of elite performance in soccer^5,6^. These top-down processes underpin higher-order cognitive processes, enabling control and regulation of thought and behaviour^7^.

Due to EFs significant development during early and middle adolescence^7–9^, they are considered as potential predictors of talent and elite performance in soccer^2,3^. While research suggests links between EFs and expertise^10,11^ or game-relevant constructs such as game intelligence^12^, questions about their validity and predictive value persist^13^. Many previous studies rely on correlational data, with a lack of longitudinal or prognostic analyses needed to establish causality^14,15^. In addition, research on elite players shows significant interindividual variability within samples and developmental patterns resembling those in the general population^13,16^. This prompts deeper exploration of whether elite youth players inherently possess superior EFs or develop them through gaining higher levels of experience^5,13,15^, and whether these functions can significantly contribute to senior performance in a complex and dynamic environment.

In this article, we explore several interconnected topics concerning the role of EF in elite youth soccer, specifically the association between EFs and coaches’ evaluation of players’ potential, their age-related development, and their predictive value of future senior performance levels.

## Executive functions in sports

Core EFs, including inhibition, working memory, and cognitive flexibility^17^, underpin higher-order cognitive processes such as reasoning and planning. Their role in controlling and regulating thoughts and behavior makes them central to effective decision-making^17,18^. The unity and diversity framework^19^ characterizes EFs as distinct yet interconnected, emphasizing their separable but interdependent nature^8,9^. Specifically, inhibition is considered as a fundamental component of both working memory and cognitive flexibility (unity), while the latter functions also encompass updating and shifting, respectively, reflecting aspects of diversity^19,20^.

This study focuses primarily on cognitive flexibility and inhibition, both of which are particularly crucial in soccer due to dynamic and unpredictable nature of the game^21^. Inhibition allows players to focus their attention on relevant cues and suppress ineffective actions, such as avoiding a pass to a tightly marked teammate. Cognitive flexibility enables the rapid integration of new information, such as changes in ball possession, and facilitates adaptive behavioral adjustments. Well-developed EFs may therefore confer a competitive advantage, enhancing technical execution and tactical decision-making^6,22^.

### Age-related development of EFs

The development of EFs is closely tied to the maturation and growth of brain regions, particularly the prefrontal cortex^19,23^. Significant improvements occur between childhood and early adolescence, with the potential to reach adult-like performance levels as early as ages 12 to 15^7,24^. This period is followed by a slower yet continuous improvement up to the age of 18, after which EF performance plateaus^7,13^. Thus, adolescence represents a critical phase for EF development, reflecting the maturation of the underlying brain regions^7,25^. Research on youth elite soccer players suggests their progression mirrors that of the general population^13,26^. A recent cross-sectional study indicated a pivotal turning point in EFs around the age of 11^27^, potentially linked to a shift in decision-making strategies^28^. This aligns with evidence showing that activation of task-relevant neural networks such as the prefrontal cortex increases with age, while activity in non-involved areas decreases^29,30^. Therefore, EF performance during adolescence appears to be shaped by chronological and biological age, reflecting individual differences in brain maturation^31^.

## Measuring EFs in sports

EFs have gained growing attention in the context of talent identification in soccer. Numerous studies have demonstrated superior performance in elite players compared to amateurs, or in athletes selected versus non-selected for promotion stages, suggesting that EFs may contribute to distinguishing higher-potential players from their peers^5,10,32^.

In sports, research on cognition has primarily followed two approaches: the cognitive component skills approach^33^ and the expert performance approach^34^. The former posits that athletes possess superior general cognitive skills, assessed with general computer-based cognitive tasks, such as the Stroop and flanker tasks (inhibition), the n-back task (working memory), and the number-letter task (cognitive flexibility). Empirical studies have shown that athletes often excel in aspects such as processing speed and attention^18,35^. However, sports require specific cognitive skills tailored to each sport, a nuance overlooked by the cognitive component skills approach^34^. Consequently, a meta-analysis across sports found no evidence that general cognitive testing could predict future sports performance^36^.

The expert performance approach suggests that athlete’s exhibit enhanced sport-specific cognitive skills. Therefore, athletes are tested in sport-specific contexts, such as analyzing sport-related images or videos. This approach has been used to investigate perceptual-cognitive skills such as attention, anticipation, and decision-making, with research indicating that athletes outperform non-athletes in these tasks^37^. To further enhance the representativeness of diagnostics tests of cognitive skills some studies incorporate standardized sport-specific motor responses^38,39^.

Despite extensive evidence of elite players’ superiority in general and sport-specific EF tasks, as well as correlations with soccer-specific motor skills, the predictive value of EFs soccer remains unclear. It is particularly debated whether superior EF levels in youth players reflect accumulated sporting experience and expertise or are primarily influenced by genetic factors^13,40^. Moreover, isolated measures of perception and information processing are insufficient performance indicators in soccer, as successful outcomes also involve physiological and motor-technical skills^41^. Consequently, a key challenge for research is to accurately assess cognitive skills within soccer-specific contexts and to investigate their predictive value within the sport’s multidimensional performance structure^15,42^. Therefore, we follow calls for longitudinal designs and for evaluations of EFs prognostic relevance in the context of soccer^14,15,43^.

## Aims and objectives of the present study

In this study, we aim to thoroughly explore (i) whether EFs can explain coaches’ evaluations of the players’ future potential, (ii) the age-related development of EFs, and (iii) the predictive value of EFs for senior performance levels in a cohort of elite youth soccer players aged 13 to 18. Analyses of (iii) are further divided into iii_a_ (i.e., first year on senior level) and iii_b_ (i.e., five years after initial tests) to address the need for different and meaningful prognostic periods^15,43^. Building on the successful transfer and validation of general computerized tasks for measuring cognitive flexibility and inhibition in a soccer-specific context^44^, we aim to incorporate tasks with a more soccer-specific perception-action coupling^15^ and compare the contributions of both tasks to explaining soccer performance. To obtain a comprehensive understanding, we employ different dependent variables as criteria for assessing talent and performance levels. These include subjective measures, such as coaches’ evaluations of players’ potential (i), and objective outcomes, such as the senior league levels attained (iii_a_, iii_b_).

Regarding the association between EFs and coaches’ evaluations (i), we hypothesized that players with higher EF performance will be rated by coaches as having greater potential to reach higher levels in senior soccer. Regarding longitudinal development (ii), we expect improved performance with age. Based on evidence indicating higher validity of domain-specific tests for distinguishing between higher and lower-skilled athletes^36^, we further expect that the sport-specific tasks will provide better predictive value (iii_a, b_). Additionally, in line with research on the age-related development in EFs, we hypothesize stronger predictability for older players, as recently demonstrated across various domains of juvenile athletic performance^45^.

## Methods

### Participants

**Fig. 1:**
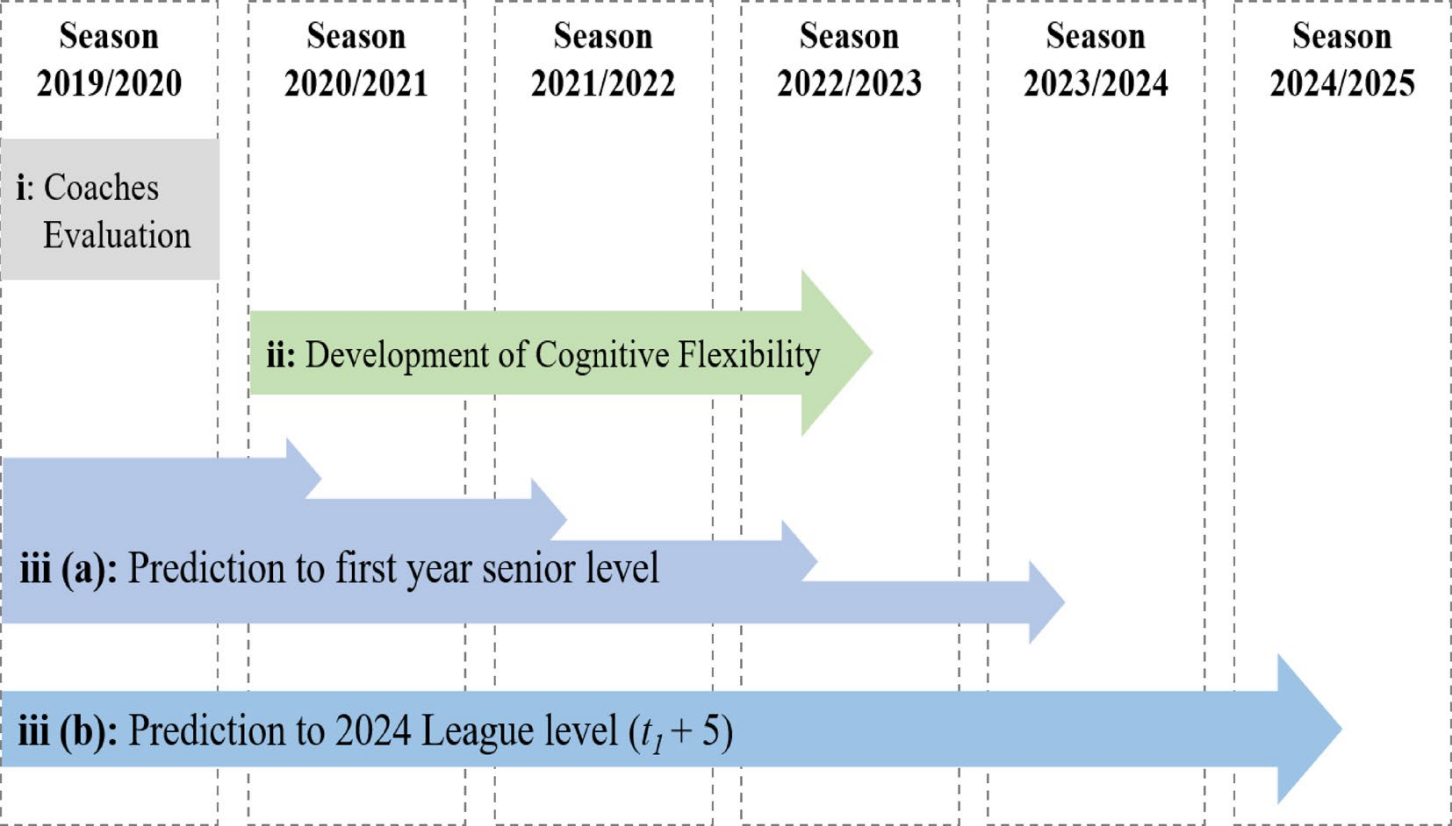
Overview of data collected over time and corresponding research questions. EF data on computerized and soccer-specific inhibition and cognitive flexibility has been previously published^44^.

The EF data analyzed in this study originates from the 2019 preseason (*t1*), involving 77 youth soccer players from an elite youth academy in Germany (a priori analysis in G*Power (Erdfelder et al., 2009) revealed a minimum required sample size of 63 participants for the original study^44^. During this period, we administered both computerized tasks and newly developed tasks within the SoccerBot360 system to assess inhibition and cognitive flexibility. All players were members of the U15, U16, U17 and U19 youth teams (*M*_age_ = 15.7, *SD*_*age*_ = 1.3), competing at the highest level of their respective age group. Due to numerous factors such as injuries, transfers or players leaving the academy after U19, longitudinal data was not feasible for 43 participants from the initial data collection. Therefore, for the longitudinal analyses (ii), we used data from 34 participants who were repeatedly assessed with the adjusted version of the number-letter task in the SoccerBot360. The first measurement point (*t*_1_; *M*_age_ = 14.5, *SD*_age_ = 1.02) was conducted during the preparation for the 2020/2021 season. The second measurement point (*t*_2_; *M*_age_ = 16.4, *SD*_age_ = 1.02) occurred two years later, during the preparation for the 2022/2023 season (see Fig. 1). Based on the original study (i), which was designed under careful a-priori power calculations, the players were followed up longitudinally (ii, iii). The final sample consisted of all eligible and available players who voluntarily agreed to participate at the time of data collection, resulting in complete data sets of (i) *n* = 74, (ii) *n* = 34, and (iii) 69 players (see data preparation and analyses for more details).

Prior to the investigation, we obtained written informed consent from the participants and their legal guardians. The study was carried out in accordance with the Declaration of Helsinki and approved by the ethics committee of Leipzig University (2020.11.17_eb_69).

### Material

#### SoccerBot360

In the initial study^44^, we transferred computerized tasks in the SoccerBot360 (SB), a 10-meter diameter circular training device with a 90-m² field and a 32-segment wall for projecting training content and interacting with the ball. This setup, featuring six high-definition projectors and an integrated high-speed camera, creates a 360° environment and simulates a soccer field with artificial grass. It also enables the projection of soccer-specific stimuli (e.g., players, goals) and measures soccer-specific motor responses (e.g., passes, shots) using parameters such as response time, ball speed, and accuracy (see supplementary figure S1 and detailed description in Section A).

#### Inhibition

An adapted version of the flanker task^46^ measured inhibition. Instead of arrows, the SB task used five side-view soccer players, with the middle player as the target flanked by two distractors on each side (see supplementary figure S2). Players pressed a white or black key in the computerized task or kicked the ball left or right in the SB task, based on the middle player’s direction. In congruent trials, all players faced the same direction, while in incongruent trials, the target and flankers faced opposite directions. The validation included four practice and 144 test trials (96 congruent, 48 incongruent) in the computerized task, and 108 (72 congruent, 36 incongruent) in the SB task^47^. Response times (ms) and accuracy (%) were recorded, and the flanker effect was calculated by subtracting congruent from incongruent trials.

#### Cognitive flexibility

Cognitive flexibility was assessed using an adapted version of the original number-letter task^19,48^. In both computerized and SB versions, participants viewed a 2 × 2 matrix (see supplementary figure S2) with a number-letter pair (e.g., 4I or A7) in one of four quadrants. When the pair appeared in the top quadrants, participants responded to the letter, pressing white for consonants and black for vowels. When in the bottom quadrants, they focused on the number, pressing white for even and black for odd. In the SB task, responses were made by passing into the left or right goal instead of pressing keys. Non-switch trials kept stimuli in the same row, while switch trials required shifting focus between letters and numbers. After 24 practice trials, the computerized task included 128 test trials (64 switch, 64 non-switch) and the SB task had 112 (56 each). After successful validation of the SB task, the trial number was reduced by half. Response times (ms) and accuracy (%) were recorded, with switch costs calculated as the difference between switch and non-switch trials.

### Coaches’ evaluation

Coaches’ subjective assessments have been demonstrated to be potentially an economical, reliable, and valid method for evaluating players’ potential and predicting their future performance^49,50^. Eight to ten weeks after the initial validation study, the sport psychologist of each respective club invited the coaches to assess their players. Coaches were asked to predict the level the player would reach by adulthood, categorizing them into one of the following four categories: (A) top player (professional player at Champions League level), (B) professional player (1st, 2nd, or 3rd German league, 1st league abroad), (C) 4th league and below, or (D) 6th league and below. Therefore, the coaches’ assessments focused not primarily on current performance but rather on the perceived potential of the player. This evaluation of the club’s academy players was integrated into the routine operations of the academy. Consequently, the question and answer categories were not designed by the research team but were established by the individuals responsible within the academy. The academy’s sport psychologists then provided access to these assessments for the study.

### Assessment of senior performance levels

To assess the prognostic value of the cognitive tasks, we followed the career progression of all players who participated in the initial tests 2019. The league in which players compete serves as a performance criterion, categorizing them into different groups according to their league level. Based on the coaches categorization, the league levels were classified as “elite” for category A and B, “sub-elite” for category C, and “non-elite” for category D^51,52^.

### Procedure

The coaches’ evaluation of the players’ future potential (i) was collected eight to ten weeks after the initial validation study, without informing the coaches of the cognitive test results. Following the validation study, we made slight adjustments to the cognitive flexibility task by reducing the number of trials by half. The inhibition task was completely revised due to unacceptable convergent validity^44^. In the subsequent years, we tested the U15 to U19 teams annually during preseason through 2022. However, the revision and validation of the flanker task in the SB were not successfully completed until 2021. Therefore, we will focus on cognitive flexibility for the longitudinal analysis (ii).

**Fig. 2:**
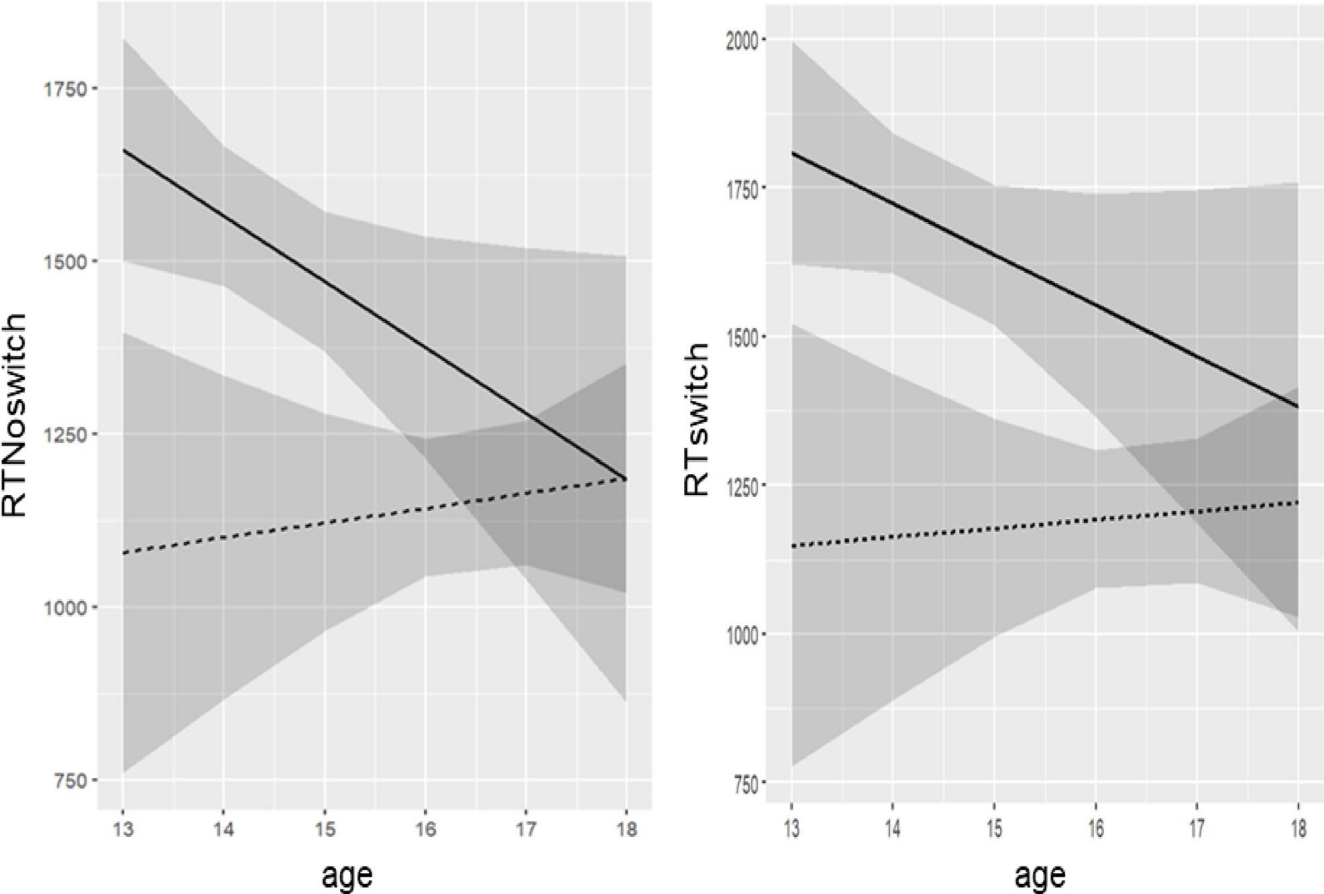
Predicted reaction times in no-switch trials (ms) as a function of measurement time and age, interaction plot. *t*_1_ = ——; *t*_2_ = -------. The grey zones surrounding the *t*_1_ and *t*_2_ mean response times across age represent the 95% confidence intervals. Therefore, the overlapping of the grey zones represents the ages where response time differences between *t*_1_ and *t*_2_ were nonsignificant.

At the start of the 2024/2025 season, with the assistance of the club and additional searches in online databases (e.g., Transfermarkt), we tracked the players’ transitions to the senior level. Given the diverse age range of players at the time of testing, we utilized two distinct dependent variables to indicate future success and expertise. Firstly, we evaluated players’ performance level in their initial year with a senior team (iii_a_). Secondly, we collected their performance data five years after the initial test, in 2019, at the start of the 2024/2025 season (iii_b_). By this time, all players had transitioned from the youth academy to the senior level. The cut-off date for current club affiliation was 2024, July 30. Figure 2 provides a comprehensive conceptual overview of the collected data, along with the research questions and the analyses conducted based on that data.

### Data preparation and analyses

The statistical analyses were conducted using SPSS (version 29) and R (version 4.4.1). A two-tailed significance level of *p* = .05 was applied to all statistical tests. As indicators of cognitive performance, we utilized response times and accuracy across the respective task conditions (i.e., congruent, incongruent; non-switch, switch). The switch costs and flanker effect were excluded from the analyses to avoid redundancy, as their calculation overlaps with the mean values of the parameters already included. Additionally, difference scores are less effective for individual comparisons because they do not account for global performance levels and potential speed-accuracy trade-offs^53^. In other words, participants may have significantly slower response times compared to others but demonstrate lower switch costs if they respond more consistently in both conditions.

#### Coaches’ evaluation

For the cognitive tasks assessing inhibition and cognitive flexibility, we only included players with complete data sets in the computerized and in the soccer-specific tasks. Because the dependent variable reflected discrete performance levels rather than a continuous outcome, a multinomial logistic regression was employed. This analytical approach is well suited for categorical outcomes, as it models non-linear relationships and does not assume normally distributed predictors^54,55^. Data were collected from all available and voluntarily participating academy players (*N* = 77); however, three players were excluded from further analyses due to missing data in one or more tests, resulting in a final sample of 74 players. To address multicollinearity among correlated EF indicators (e.g., no-switch, switch, switch costs), a principal component analysis (PCA) with oblique Promax rotation was conducted, allowing for correlations between factors^54^. This analysis extracted four factors (Factor 1: response times for switch and no-switch trials in the SB and PC tasks, as well as SB accuracy; Factor 2: response times for congruent and incongruent trials in SB and PC; Factor 3: accuracy in congruent and incongruent trials for SB and PC; Factor 4: accuracy in switch and no-switch trials in PC). These factors extracted from the original variables were then used as predictor variables in the regression analyses (for further details, see supplementary table S1). Although PCA primarily aims at variance reduction, the resulting components were interpreted as latent dimensions reflecting underlying executive functions, consistent with the latent variable approach used to study EF structure in previous research^19^. This procedure helped to reduce redundancy, mitigate multicollinearity, and improve interpretability, while reflecting the practical constraints of elite talent research and maintaining ecological validity.

#### Age-related development of cognitive flexibility

For the 34 participants who completed the measurements on cognitive flexibility in the SB in 2020 and 2022, we performed a multilevel analysis in R (lmerTest). In detail, a mixed-effects model with fixed effects for the predictors age and measurement time was modeled. Age was applied as a continuous between-subjects variable and measurement time (two levels: *t*_1_, *t*_2_) as within-subjects variables. In a step-wise manner, we compared different models for each dependent variable separately. We compared a random intercept for the subject (i.e., allowing individual starting points) with a random intercept and random slopes model that contained either only main effects or the interaction terms in addition. This model comparison approach was followed for each of the three different dependent variables: (1) response times no-switch trials; (2) response times switch trials; (3) overall accuracy (hit rate).

#### Predictive value of EFs

We conducted additional multinomial logistic regressions using the league achieved in the players’ first senior year (iii_a_) and the league they are competing at the beginning of season 2024/2025 (iii_b_). For eight players, their first senior year coincided with the current season. From the 74 players with complete datasets for analysis (i), five were excluded due to unclear classification of their current leagues or because they were not actively playing at the club level, leaving a final sample for this analysis of 69 players. Similar to the analyses on the coaches’ evaluation, we ran the analyses with the four extracted factors and age as predictor variable. Additionally, we included coaches’ evaluations as a factor to assess their potential impact on player development, such as increased playing time.

## Results

Descriptive statistics for the tests conducted across all participants can be found in the supplementary table S2.

### Coaches’ evaluation

The multinomial logistic regression model, which included the four extracted factors and age, was not significant, χ²(10) = 8.439, *p* = .586, Nagelkerke’s *R*² = 0.122. Also, each individual predictor variable showed no significant relation to the future performance level evaluated by coaches (all *p* ≥ .125, see supplementary table S3). This result indicated that there was no meaningful statistical relation between the EFs assessed and the subjective, prospective coaches’ evaluation of the player’s future performance level.

### Age-related development of cognitive flexibility

Longitudinal analyses, which were conducted for cognitive flexibility, revealed that the random intercept and slope model including interaction terms resulted in the best model fit for response times in the no-switch condition (*R*^2^ = 0.34). The model revealed a significant main effect of age on response time (*B* = -153.66, *p* = .012) showing that older players responded faster in the no-switch condition. The time of measurement was also a significant predictor, *B* = -1049.96, *p* = .004. In particular, the analysis showed that response times decreased from *t*_1_ (*M* = 1517.82, *SD* = 344.79) to *t*_2_ (*M* = 1152.76, *SD* = 203.22). The main effects were further qualified by the significant interaction between age and time of measurement, *B* = 58.38, *p* = .011, indicating that the effect of age on reaction time depends on the measurement time (see Fig. 2). As shown in the interaction plot, until the age of 15.7 players improved their response times in the no-switch trials significantly from *t*_1_ to *t*_2_, while they did not improve significantly above that age anymore.

For response time in the switch-condition, the random intercept and slope model with interaction terms similarly resulted in the best model fit (*R*^2^ = 0.38). While age had no significant main effect on response times, *B* = -135,42, *p* = .065, measurement time was a significant predictor, *B* = -979.11, *p* = .030. As observed in no-switch trials, response times were reduced from *t*_1_ (*M* = 1680.60, *SD* = 410.27) to *t*_*2*_ (*M* = 1199.29, *SD* = 209.35). Again, the main effects were further qualified by the significant interaction between age and time of measurement, *B* = 49.96, *p* = .030 (see Fig. 2). As shown in the interaction plot, until the age of 16.2 players improved their response times in the no-switch trials significantly from *t*_1_ to *t*_2_, while they did not improve significantly above that age anymore.

For accuracy, the random intercept and slope model without interaction terms indicated the best model fit (*R*^2^ = 0.13). Age (*B* = 0.01, *p* = .334) and measurement time (*B* = 0.03, *p* = .141) showed no significant effects.

### Predictive value of EFs

The regression model for the first senior level (iii_a_) was not significant χ²(14) = 18.990, *p* = .165, Nagelkerke’s *R*² = 0.277. Except for the “non-elite” coaches evaluation category (C = “non-elite”), *B* = 2.66, *p* = .046, OR = 14.26, none of the predictors did significantly explain variance of first-year senior league levels (see supplementary table S4).

The model for the current league level in the 2024/2025 season also failed to demonstrate predictive validity of the cognitive variables χ²(14) = 13.946, *p* = .454, Nagelkerke’s *R*² = 0.219, with no predictor being significant (see supplementary table S5). All regression analyses were repeated without principal component analysis, incorporating the individual variables directly. These included two separate analyses for each measure: response times and accuracy, for both cognitive flexibility and inhibition. The regression analyses containing all dependent variables showed the same pattern of results.

## Discussion

This study investigated whether the core EFs of inhibition and cognitive flexibility can contribute to predicting the career progressions of youth elite soccer players. Specifically, we examined whether EF data obtained in computer-based and soccer-specific settings could account for both subjective (i.e., coaches’ assessments of talent and future performance) and objective (i.e., attained senior levels) performance parameters. Additionally, we tracked players’ cognitive development over a two-year period, considering the influence of age and measurement point. Contrary to previous findings that reported positive associations between EFs and performance, the results of this study do not support a relationship between EFs and (future) soccer performance. However, these findings contribute to the ongoing debate about the role of EFs in (elite) sports.

### Executive functions and coaches’ evaluation

Our analyses showed no statistically significant relationship between individual EF performance, measured with general computerized and the adapted SB tasks, and coaches’ evaluation of future soccer performance. The direction of associations was inconsistent, with non-significant trends suggesting both enhanced (e.g. factor 3, OR = 0.560) and diminished (e.g. factor 4, OR = 1.554) cognitive performance among elite ranked players compared to non-elite ranked players (see Supplementary Table S3). These findings contrast with previous research showing a relationship reporting a link between coaches’ ratings and design fluency performance, although that study focused narrowly on game intelligence ratings^40^. Correlations have likewise been observed between game intelligence ratings and EFs^12^, suggesting that closer-defined performance constructs may be more likely related to EF performance. Another difference lies in the sample, as the other studies included adult players, where coaches’ evaluation may be more reliable than in youth contexts. Besides, numerous confounding factors influence the development of soccer performance, limiting the accuracy of subjective evaluations. Subjective talent assessments in soccer often focus on aspects such as athleticism, agility, and technical-motor skills^56,57^. Thus, in the youth stages, chronological and biological age, along with the resulting developmental advantages, often influence talent selection and the respective prognoses^58^. Consequently, cognitive abilities may play a lesser role in coaches’ evaluations compared to directly observable attributes of players. In line with this, compensation effects have been proposed in talent research, whereby players might counterbalance lower cognitive abilities with exceptional speed and agility^42,59^.

### Longitudinal development of cognitive flexibility

Multilevel analyses of cognitive flexibility measured with the SoccerBot revealed varying age-related changes in EFs. Cognitive flexibility, regarded as the most complex core EF^19^, is typically reported to reach adult-like levels by the age of 15^7,23^. Our results confirm this trajectory, with significant interactions between age and measurement time. Younger players (ages 13–15) displayed greater fluctuations and changes across the two-year period than older players (ages 16–18). This aligns with age-related trends observed in both general and athletic population^7,26,31^. Additionally, the findings offer insights into the developmental trajectory of flexibility demands, as closer inspection revealed distinct developmental cut-offs. Response times in less complex no-switch-trials improved until age 15, whereas improvements more complex switch-trials continued until age 16. This pattern suggests that higher switching demands remain challenging for adolescents and may therefore serve as a potential indicator to differentiate between higher- and lower-skilled athletes. For the applied setting, the strong variability before age 15 limits the diagnostic value of EF diagnostics in younger age groups. However, due to the lack of normative or reference values from other athletes or performance levels, the results do not allow for conclusions about the development of EFs in relation to sports experience and performance^47,60^. Further, in the regression analyses predicting future performance the age at *t*_1_ did not have a significant influence in any of the regression analyses.

### Executive functions and senior performance levels

EF are widely debated in relation to future soccer performance with meta-analyses drawing divergent conclusions^35,36^. In the present study, EFs did not significantly predict senior performance levels, regardless of the time period examined (i.e., first year on senior level or five years after testing). Instead, coaches’ evaluation, particularly categorizations as “non-elite” (OR = 14.264, *p* = .046), appeared more informative, although wide confidence intervals (1.054–193.126) highlight considerable uncertainty (see Supplementary Table S4). These findings diverge from studies reporting positive relationships between EFs and expertise in soccer^11,32,61^, but align with others showing no predictive value^13,36,62^. Thus, our results may help to further address a possible publication bias that has been previously highlighted in the research area of EFs in sports^14,36^. More broadly, given the complex and multifaceted nature of high performance in soccer, considering single aspects in isolation appears insufficient for predicting future success^2,15,63^. This applies not only to EF measures but also to tests of individual athletic, motor and psychological attributes^64,65^. Furthermore, generalizations across players are further complicated by positional demands as roles, objectives and performance requirements differ substantially within a team^40^.

Methodologically, neither the general measure of cognitive flexibility and inhibition nor the adapted SB tasks demonstrate significant explanatory value, despite previous findings suggesting better differentiation with sport-specific tests^36^. Despite their specific response modalities and context, our tests remain insufficiently representative of the complex demands on the field. Additionally, test outcomes may also be influenced by other variables such as motivation, task utility, enjoyment, or experience^14,36^. In this context, testing under neutral conditions may also weaken the connection with actual soccer performance, since competitive matches involve stressors known to alter EFs^16,66^. Therefore, advancing representative testing methods and incorporating competitive conditions are essential steps for further understanding the role of EFs in the development of athletic performance^67,68^. Based on our findings, we also question using EF measures as reliable indicators for talent identification, selection and prediction processes^13,14^.

### Limitations

This study has several limitations that could inform directions for future research. First, the absence of comparison groups, such as amateur players or elite level adults, limits the ability to contextualize our findings. Second, the specificity of the sample – elite youth players from one club – naturally restricted the participant pool, yielding a relatively small sample size. Although typical in expertise research^69,70^ this may have contributed to non-significant results. Future studies should therefore aim to collect cognitive data from larger samples, for example across different academies and institutions, similar to multi-center studies in the clinical setting^71^. Third, as the SB is a relatively new instrument, normative data across different age groups and performance levels are lacking. Collecting such data and establishing benchmarks could provide valuable insights into whether specific patterns emerge across various groups and categories. Forth, we did not assess the full range of EFs. Relying on two tasks may insufficiently capture the diverse components involved. Future research should employ a broader range of tasks and scoring metric. Finally, the high correlations between cognitive variables suggest that the tasks capture the interaction between inhibition and cognitive flexibility more effectively than isolating specific aspects of EFs. In this context, it is also important to reiterate that the version of the flanker task used in the SB did not show a strong correlation with the PC test and was subsequently adjusted.

## Conclusion

This study examined whether the core EFs of inhibition and cognitive flexibility can account for subjective (coaches’ evaluation) and objective (adult performance level) future performance metrics in youth elite soccer players. In addition, we tracked the cognitive development of players that remained in the academy system over a two-year period, considering the influence of age and measurement point. The present findings in this sample show that EF performance was not associated with coaches’ evaluation and future soccer performance levels, while cognitive flexibility showed age-related developmental trends similar to those in the general population. These findings contribute to previous research on the role of EFs in elite youth sports, their possible relation to future, senior performance levels in soccer, and suggest areas for further investigation. Future research should aim for larger elite samples, employ a broader range of tasks and scoring metrics to capture the complex nature of cognitive performance, while incorporating comparison groups and more contextually relevant testing conditions. Overall, our findings suggest that EFs alone may not be sufficient to predict soccer performance, underscoring the need to integrate cognitive data into multifaceted approaches to talent identification and development.

## Data Availability

All data, exclusions, manipulations, procedures, ethical guidelines, and other methods developed by the authors are fully documented and cited within the text. Due to the sensitive nature of the data and to protect the anonymity of the youth players, the datasets generated and analyzed cannot be made publicly available. However, data can be shared upon reasonable request to the first authors.
